# Hip and Knee Bilateral Deficit Across Bilateral, Unilateral, and Split-Load Leg Press Conditions

**DOI:** 10.3390/jfmk11020216

**Published:** 2026-05-28

**Authors:** Anna Pisz, Dusan Blazek, Petr Stastny

**Affiliations:** 1 Department of Sport Games, Faculty of Physical Education and Sport, Charles University, 16200 Prague, Czech Republic; petr.stastny@ftvs.cuni.cz; 2 Department of Track & Field and Outdoor Activities, Faculty of Physical Education and Sport, Charles University, 16200 Prague, Czech Republic; dusan.blazek@ftvs.cuni.cz; 3 Institute of Sport, Akademia Wychowania Fizycznego im. Jerzego Kukuczki w Katowicach, 40-065 Katowice, Poland

**Keywords:** bilateral deficit, leg press, limb asymmetry, isokinetic dynamometry, lower-limb strength

## Abstract

**Objectives**: This study investigated bilateral strength asymmetry, the bilateral asymmetry index, and the bilateral deficit ratio during one-repetition maximum leg press testing performed under bilateral, unilateral, and split-load conditions and examined their associations with isokinetic knee and hip strength asymmetries. **Methods**: 31 resistance-trained males completed 1RM leg press tests in all loading modes, followed by isokinetic knee flexion/extension and hip extension assessments at 60°·s^−1^. **Results**: The repeated measure ANOVA showed that split-load leg press had a significantly greater bilateral deficit ratio (14.29 ± 7.71%) compared to the bilateral condition (5.16 ± 9.60%, *p* < 0.001). Isokinetic testing showed significant inter-limb differences for knee flexion and extension but not hip extension. The bilateral strength asymmetry varied across tasks (5.17 ± 4.44% in leg press to 17.84 ± 12.40% in eccentric hip extension), while bilateral asymmetry index remained consistently lower. Bilateral strength asymmetry differed significantly across leg press conditions, whereas the bilateral asymmetry index did not. Knee flexion bilateral asymmetry index correlated with dominant and non-dominant hamstring to quadriceps ratios (respectively, r = 0.61; r = 0.37) and cross-limb flexor–extensor ratios (r = 0.42). No significant relationships were found for hip extension asymmetry. **Conclusions**: Split-load leg press might be used to test lower limb bilateral deficit, because it provides easily detectable deficit values. Unilateral leg press might be used to detect lateral strength differences, since it provides relation to isokinetic strength of knee flexors and extensors.

## 1. Introduction

Lower-limb asymmetry is a multifaceted, task-specific attribute of athletic performance and injury risk. The literature consistently shows substantial heterogeneity in measurement methods, injury definitions, follow-up durations, and sport-specific demands, which undermines the utility of universal risk cutoffs. Consequently, sport- and task-specific asymmetry profiles are recommended to guide monitoring and intervention strategies [1]. Imbalances manifest in various forms, including lateral deficits, intermuscular imbalances such as hamstrings-to-quadriceps (H/Q) strength ratios, and functional asymmetries observed in dynamic movements. The relationship between these imbalances and injury incidence is complex, modulated by sport type, movement demands, and the magnitude and distribution of asymmetry across tasks and planes (e.g., sagittal vs. frontal loading) [1].

One of the general concepts of strength asymmetries is the bilateral deficit (BLD), which refers to the phenomenon where force output during bilateral movements is less than the summed output of unilateral efforts, reflecting neuromuscular coordination and inhibitory mechanisms. Factors contributing to BLD include neural activation patterns, biomechanical stability, and psychological aspects such as perceived exertion [2,3]. A significant BLD can indicate inefficient interlimb coordination and neuromuscular inhibition, potentially limiting maximal performance output and predisposing individuals to injury due to compensatory movement patterns and muscle overloads [4], especially in the lack of unilateral strength. Additionally, inter-limb asymmetries in force production have been shown to negatively affect sprint and change-of-direction performance, while excessive postural asymmetries are connected to chronic conditions like groin pain [5,6]. Athletes with previous injuries may continue to exhibit residual bilateral net force deficits, further elevating their risk of re-injury [7]. Multi-joint tasks such as leg-press-type movements often reveal larger asymmetries than isolated joint tests, underscoring the need for ecologically valid, task-relevant assessments of strength balance [8].

While the leg press is a widely utilized tool for lower limb strength assessment, few investigations have systematically compared the traditional bilateral configuration to a split-load approach—where each limb moves independently—for their capacity to detect functional inter-limb strength asymmetries. Exploring whether the split-load technique offers improved sensitivity for uncovering subtle neuromuscular differences remains an important research need. Leg press exercise is particularly versatile, as it can be performed in bilateral, unilateral, or split-load modes, accommodating a range of assessment strategies, e.g., the unilateral leg press allows for calculating functional lateral strength differences such as bilateral strength asymmetry (BSA) or BLD. It is also considered a safer alternative to squats, especially for individuals with back issues or limited mobility, due to its enhanced support and stability [3,9]. Furthermore, variations in foot placement and single-leg execution enable athletes to address and potentially correct muscular imbalances, supporting balanced lower body development [10]. Given its accessibility and safety, the leg press stands as a promising alternative to traditional laboratory-based tools such as isokinetic dynamometry. Nonetheless, to establish its practical value in identifying muscle deficits or monitoring performance, it is necessary to directly compare its effectiveness to that of isokinetic testing.

Previous research on BLD has shown that its magnitude can vary depending on the type of exercise and muscle group involved. BLD tends to be more pronounced in compound, lower-body exercises such as the leg press and half-squat compared to isolated movements like handgrip exercises, likely due to increased postural demands and neuromuscular coordination requirements in multi-joint tasks [11]. For example, in lower-limb isometric and dynamic contractions, bilateral deficits of 6% and 37% have been commonly reported, particularly when high-velocity or explosive strength is tested [12]. Several factors contribute to these effects: alterations in muscle excitation at both spinal and supraspinal levels [13], greater postural stability in unilateral compared to bilateral tasks [14], a downward shift in the force–velocity relationship during unilateral efforts [15], impaired coordination among synergistic muscles [15], and reduced effort perception in unilateral compared to bilateral contractions, particularly in lower limb muscles [16]. Some populations, such as novice or untrained individuals, may display larger BLDs due to undeveloped motor coordination; experienced athletes may show either reduced deficits or even bilateral facilitation depending on their training background and task specificity [17]. These findings indicate that BLD is not only a neural phenomenon but also biomechanically influenced by task complexity, stability demands, and movement velocity.

The leg-press-based strength assessment captures the aggregate output of knee extensors, hip and ankle contributions, and coordinating muscles as engaged by multi-joint leg-press movement. This aligns with findings indicating that interlimb asymmetries observed in multi-joint tasks (e.g., leg press) can differ in magnitude and directional characteristics from those observed in isolated isometric or isokinetic knee-extensor tasks. Šarabon et al. emphasize that interlimb asymmetry magnitudes can be large (>20%) in maximal strength for bilateral tasks, particularly in leg-press-type movements, and that such asymmetries reflect task-level organization rather than solely single-joint limitations [18]. This support using leg-press-based strength measurements as ecologically valid indicators of functional strength asymmetry in daily activities and sport.

Although previous studies have confirmed the presence of BLD in leg press and other multi-joint lower-body tasks, few investigations have explicitly examined whether hip- and knee-dominant muscle groups contribute differently to bilateral deficit within the same leg-press protocol. Therefore, this study investigated bilateral strength asymmetry, the bilateral asymmetry index, and the bilateral deficit ratio during one-repetition maximum leg press testing performed under bilateral, unilateral, and split-load conditions and examined their associations with isokinetic knee and hip strength asymmetries. Differentiating these contributions could provide clinically meaningful insights to inform targeted unilateral or split-load interventions aimed at improving inter-limb coordination and reducing injury risk during high-demand athletic actions such as sprinting, jumping, and change-of-direction tasks [2].

We hypothesized that the split-load leg press would be more effective than the traditional bilateral configuration in revealing inter-limb neuromuscular asymmetries. Additionally, we expected that asymmetry indices derived from the split-load leg press would correlate with isokinetic measures of hip and knee strength symmetry. Finally, we anticipated that the magnitude of the bilateral deficit would differ between knee- and hip-dominant muscle groups.

## 2. Materials and Methods

### 2.1. Experimental Approach to the Problem

This cross-sectional repeated measure consisted of one familiarization session and two randomly selected exercise sections.

**Figure 1:**
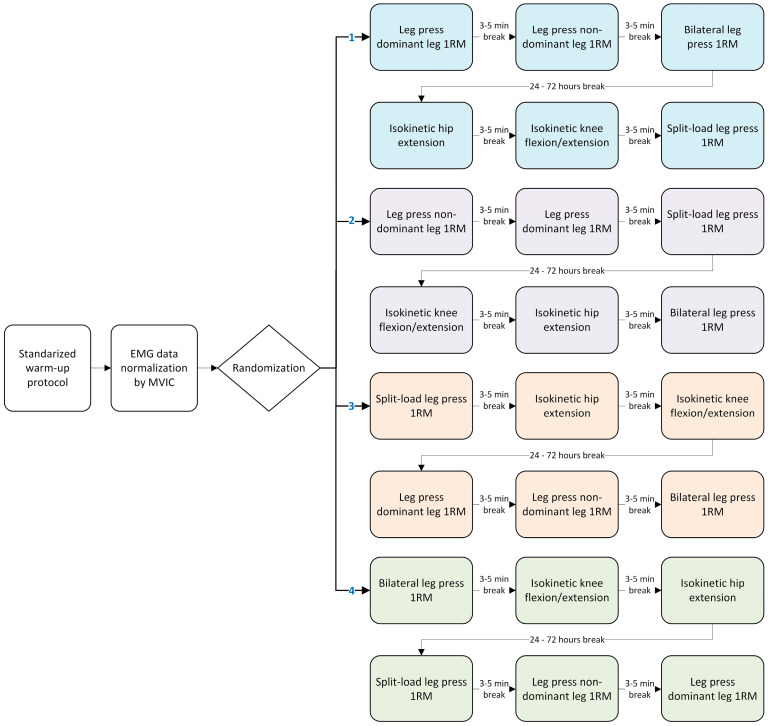
Schematic representation of the study design.

The familiarization session included basics anthropometry measurements, performing of 45° inclined leg press variations with 2 sets of 3 repetitions of each method, instructing of methodology, and signing informant content. The experimental sessions were performed in a randomized order, with minimum 48 h and maximum 72 h apart, where each participant performed unilateral leg press with dominant leg, unilateral leg press with non-dominant leg, bilateral leg press, split-load leg press with both legs moving simultaneously, isokinetic hip extension with concentric movement, hip extension with eccentric movement, knee flexion, and knee extension (Figure 1). All sessions were performed at a similar time, mostly in the morning.

### 2.2. Subjects

The sample size was calculated a priori using G*power software (version 3.1.9.3, Dusseldorf, Germany) for repeated measures ANOVA within interaction with statistical power of 0.9, a level of significance of 0.05, and effect size d = 0.25. The selected effect size was based on the findings reported by Jones [19], who observed a comparable magnitude of effect in a similar population and experimental design. The analysis indicated that a minimum of 28 participants was required.

A total of 31 resistance-trained males participated in this study (age: 21.98 ± 4.21 year; body mass: 88.5 ± 20.18 kg; height: 169.7 ± 21.98 cm; bilateral leg press performance 4.97 ± 1.09 kg/BW). All participants had prior experience with strength training (minimum 2 years regular training in the gym, minimum 2 times a week), including the leg press exercise, and were free from any musculoskeletal injuries for at least six months before enrolment. All participants were students of sport faculty. To ensure the accuracy of the study’s findings, participants were instructed to abstain from lower limb exercises during the study period and to maintain their regular dietary and sleep habits. Additionally, they were asked to refrain from using any supplements or stimulants for 24 h before testing.

Participation was voluntary, and individuals could withdraw from the study at any time. Before providing written informed consent, participants were thoroughly informed about the study’s benefits and potential risks. The research protocol received approval from the Ethics Committee of Charles University, Faculty of Physical Education and Sport (approval code: SK104/2024). Informed consent for participation was obtained from all subjects involved in the study.

### 2.3. Procedures

Familiarization session

The familiarization session began by measuring each participant’s body weight, height, and pelvic width, which was used for determining the width of stance on footplates. After these initial assessments, participants provided written informed consent and received a thorough explanation of the study’s methodology. This familiarization visit also included a section in the gym where participants were introduced to the leg press device to acclimate them to the exercise protocol and during which the proband tried out all the leg-press variations included in this research at a set 2-1-X-X tempo. A metronome set at 60 beats per minute (bpm) was used to guide the tempo, instructing participants to perform the eccentric (lowering) phase over 2 s, followed by a 1-s pause at 90° knee flexion. This pause aimed to eliminate the contribution of elastic energy accumulation [20]. The 90° angle was chosen because quadriceps activation at this joint position is comparable to that observed during an isolated knee extension exercise [21]. After this pause, the participant then performed the leg press movement.

During the familiarization session, adhesive tape was placed on the pipe of the inclined leg press device to mark the depth required for each participant to achieve 90° knee flexion, as measured by a goniometer. Researchers recorded the distance from the bottom of the frame to the bottom edge of the tape in centimeters, allowing for accurate repositioning before each session. This setup ensured that participants consistently reached the prescribed knee flexion angle during every repetition. During the familiarization session, participants practiced both unilateral leg press exercises and split-load exercises, focusing on independent movement of each leg. The device’s plates were allowed to move freely and synchronously, without any external weight applied, to ensure proper coordination and technique. This approach was designed to help participants familiarize themselves with the equipment and the specific movement patterns required for the study.

Experimental sessions

Each experimental session began with a standardized warm-up protocol. The general warm-up involved 10 min of stationary cycling at 100 W with a cadence of 80 RPM to promote blood flow and prepare the muscles for the upcoming resistance exercises. This was followed by a specific warm-up tailored to the exercise being performed in the session. Research indicates that combining general and specific warm-ups can enhance performance in strength assessments, such as the 1RM leg press [22].

Leg press session

If the session started with leg press, participants transitioned to the leg press machine for the specific warm-up immediately after completing the general warm-up. Participants performed the leg press exercise using the LAX Fitness Systems 45° inclined leg press machine (Boršice, Czech Republic). This machine features an inclined design, allowing users to execute the movement in a seated position. To ensure consistency and proper biomechanics, each participant’s foot placement on the footrest was standardized to match their pelvic width, with adhesive tape on footrest indicating the correct stance width. Additionally, participants were instructed to position their feet with an external rotation between 0° and 30°, aligning their heels with their glutes to maintain optimal knee alignment and muscle engagement. This setup aimed to provide a uniform starting position for all participants, facilitating accurate and reliable data collection throughout the study. Firstly, they performed eight bilateral repetitions at 50% of their estimated 1RM, which was initially calculated as 175% of their body weight [23]. If participants successfully completed the required repetitions with control and without excessive exertion, the load for the second warm-up set was increased to 70% of their 1RM, where they performed three repetitions. However, if they were unable to meet the repetition requirement, their estimated 1RM was adjusted downward, with the initial calculation reduced from 175% to 150% of their body weight. Rest intervals of 60 to 90 s were provided between warm-up sets, followed by a three-minute break before proceeding to the main trials.

The load for the first recorded trial in the bilateral leg press was determined based on the resistances used during the warm-up phase. If the initial weight did not precisely match 1RM, it was adjusted according to the participant’s subjective effort, and another attempt was performed. Between each trial, a rest period of three to five minutes was provided during which participants were instructed to walk.

For a trial to be considered valid, participants had to successfully complete the lift while maintaining a strict execution tempo of 2-1-X-X (a two-second eccentric phase, a one-second pause, and an immediate concentric push with no pause at the top). Additionally, they had to lower the platform deep enough to reach the predetermined 90° knee flexion checkpoint.

In the split-load leg press condition, each foot was positioned on an independent footplate, allowing both legs to move separately during the exercise. The weight was evenly distributed, ensuring the same load was applied to each leg. Only those trials in which both legs moved in a coordinated and symmetrical manner—without observable differences in force output between limbs—were considered valid for analysis. Symmetry was operationally defined as a difference of no more than 1 cm in lifting height between the plates, assessed using a distance stripe positioned between them. Any attempts that did not meet the strict 1RM criteria were excluded from the final data analysis.

During unilateral leg press technique, the passive leg was resting on the pipe from device. The number of attempts required to determine 1RM was recorded across all testing conditions. On average, participants required 3.5 ± 1.2 trials to establish their 1RM in the unilateral dominant leg condition, 3.25 ± 0.9 trials in the unilateral non-dominant leg condition, 4.5 ± 1.5 trials in the split-load leg press, and 3.5 ± 1.2 trials in the bilateral leg press condition.

Isokinetic session

If the session started in the laboratory with the isokinetic dynamometer, participants first completed a general warm-up. They then proceeded directly to a specific warm-up on the dynamometer before commencing the main testing protocol (Figure 1).

Firstly, the research team adjusted the isokinetic dynamometer (Humac Norm, Stoughton, MA, USA) to match each participant’s body proportions and set the appropriate range of motion for each exercise. For knee extension and flexion, participants were seated and performed movements within a 5° to 90° range. For hip extension, participants were positioned prone, with the exercising thigh secured to the lever arm and the knee flexed at 90°, aligned with the edge of the dynamometer seat; the lever and hip joint rotated from 5° to 75°. This protocol had previously been used in studies investigating optimal resistance settings for 1RM measurements, including in populations with overpronation [24], thereby ensuring consistency and reliability in data collection.

The specific warm-up began with an initial repetition at approximately 50% of the participant’s perceived maximal effort, followed by a 30-s rest. This was followed by a second trial at 70% effort and another 30-s rest, then a familiarization repetition at 100% perceived maximal effort to acclimate participants to the testing conditions. All warm-up repetitions and subsequent isokinetic measurements were performed at an angular velocity of 60°/s. After a two-minute rest interval, participants completed two measured repetitions each of knee flexion and extension (concentric actions) and two repetitions of hip extension (both concentric and eccentric actions). The peak torque of the best repetition was used for analysis.

### 2.4. Statistical Analyses

Data analysis was conducted according to a per-protocol approach, including only participants who completed all testing conditions without protocol deviations. No data imputation was performed. A flowchart of the sample selection process was added as Supplementary Materials. All statistical analyses were performed using SPSS (version 30.0, IBM, Chicago, IL, USA) and MATLAB R2023b software and were expressed as means with standard deviations (±SD). Two-way repeated ANOVA was used to determine differences between BSA, BAI, H/Q ratio and bilateral deficits, whereas bilateral net force was used either by bilateral or split-load leg press. To test associations between isokinetic and leg press asymmetries or ratios, Pearson’s product-moment correlation coefficient (*r*) was used. Normality of the data was assessed using the Shapiro–Wilk test prior to correlation analysis. Thresholds for qualitative descriptors of correlations were interpreted as trivial (0.0–0.09), small (0.10–0.29), moderate (0.30–0.49), large (0.50–0.69), very large (0.70–0.89), nearly perfect (0.90–0.99), and perfect (1.0) [25]. Two-way repeated ANOVA was used to compare dominant vs. non-dominant limb strength in isokinetic and leg press tests and across multiple conditions (bilateral, unilateral, split-load).

Bilateral strength asymmetry (BSA) has been calculated to assess symmetry between limbs in unilateral leg press. It was calculated as

$$BSA(\%)=\left(\frac{Stronger\;limb-Weaker\;limb}{Stronger\;limb}\right)\times 100$$

The bilateral deficit ratio (BDR) was used to determine the difference in force production between bilateral (both limbs together) and unilateral (each limb separately) movements. It was calculated using the following formula:

$$BDR\left(\%\right)=\left(\frac{\left(Sum\;of\;unilateral\;strength-Bilateral\;strength\right)}{Sum\;of\;unilateral\;strength}\right)\times 100$$

Additionally, the bilateral asymmetry index (BAI) will be calculated for calculating asymmetries during bilateral movement.

$$BAI\left(\%\right)=\left(\frac{\left(Dominant\;limb-Nondominant\;limb\right)}{Dominant\;limb+Nondominant\;limb}\right)\times 100$$

## 3. Results

**Figure 2:**
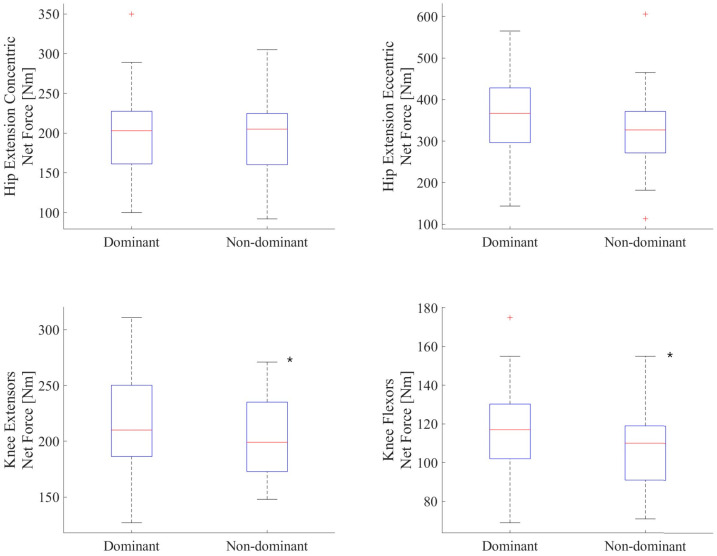
Force distribution between the limbs. * Significant difference.

Levene’s test indicated no significant violations of the homogeneity of variance assumption across all paired conditions. Table 1 shows Pairwise comparisons of BSA, across leg press and isokinetic muscle groups, whereas Table 2 shows inter-limb ratio results mean with SD. The two-way ANOVA revealed significant differences in 1RM performance between the bilateral connected and split-load press conditions F(1,30) = 49.884, *p* < 0.001, with a large effect size (partial η^2^ = 0.624). Likewise, significant difference was observed between the dominant and non-dominant legs in unilateral 1RM performance F(1,30) = 18.897, *p* < 0.001, with a large effect size (partial η^2^ = 0.386). The isokinetic assessments identified significant side-to-side differences in both knee extension and knee flexion peak torque values, favoring the dominant leg. No significant bilateral difference was found in hip extension strength under either concentric or eccentric conditions (Figure 2).

A repeated measures ANOVA revealed a significant effect of condition on BSA across leg press, knee flexion, knee extension, hip flexion, and hip eccentric actions, F(4,120) = 6.43, *p* < 0.001, with a large effect size (partial η^2^ = 0.176). In contrast, no significant differences were observed across BAI conditions, F(4,120) = 1.23, *p* = 0.303. Post hoc pairwise comparisons with Bonferroni correction showed that BSA during leg press differed significantly from BSA during knee flexion (*p* = 0.002), hip flexion (*p* = 0.022), and hip eccentric (*p* < 0.001), while other comparisons were not statistically significant (Table 1).

**Figure 3:**
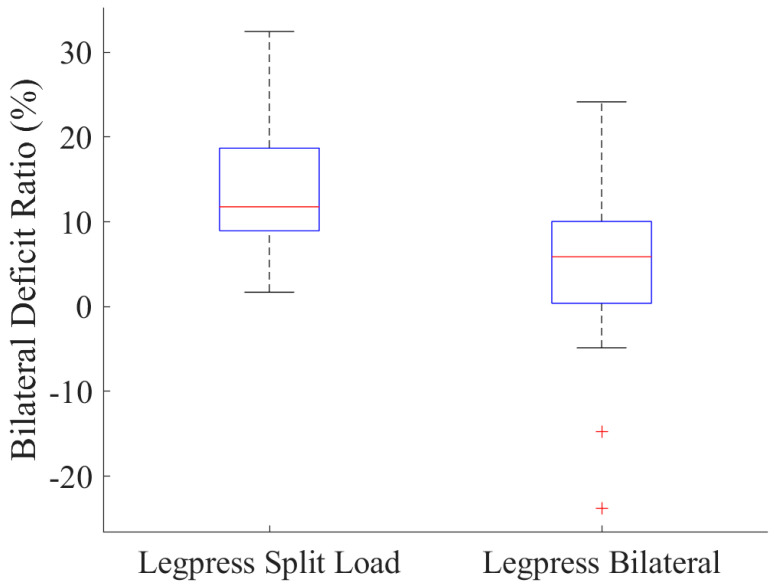
Bilateral deficit ratio on leg press.

Significant differences were observed for bilateral deficit using split-load and bilateral leg press F(1,30) = 25.94, *p* < 0.001, with a large effect size (partial η^2^ = 0.464) (Figure 3).

A moderate, statistically significant negative correlation was observed between BSA in knee extension and the H/Q ratio of the dominant leg (*r* = −0.53, *p* < 0.01). Furthermore, a strong significant positive correlation was identified between BSA and BAI in the leg press (*r* = 0.87). Pearson’s correlation analysis revealed that BAI for knee flexion was significantly associated with the H/Q ratio of the dominant leg (*r* = −0.61, *p* < 0.01), the H/Q ratio of the non-dominant leg (*r* = 0.37, *p* = 0.042), and the dominant flexors to non-dominant extensors ratio (*r* = 0.42, *p* = 0.035). BAI for knee extension was also negatively correlated with the H/Q ratio of the non-dominant leg (*r* = −0.63, *p* < 0.01). No significant correlations were found between BSA or BAI in hip extension and any H/Q or BDR values (Table 3).

A moderate positive correlation was observed between the sum of knee flexion and extension forces in the non-dominant limb and the BDR derived from the split-load leg press (*r* = 0.378, *p* = 0.036). No significant correlations were found between eccentric hip extension symmetry and any leg press metrics, suggesting that eccentric hip strength balance had limited influence on leg press symmetry or performance in this context.

For cross ratios between dominant knee extensors and nondominant knee flexors, and non-dominant knee extensors and dominant knee flexors, two-way ANOVA revealed significant differences (*p* = 0.014).

## 4. Discussion

The primary objective of this study was to evaluate whether a split-load leg press configuration offers enhanced sensitivity for detecting lower-limb strength asymmetries and bilateral deficits compared to traditional testing. Our findings supported this premise, as the repeated-measures ANOVA confirmed a significant main effect of condition on 1RM performance between the bilateral connected and split-load leg press modes. Specifically, split-load testing elicited a significantly greater bilateral deficit ratio (14.29 ± 7.71%) compared to the traditional bilateral connected condition (5.16 ± 9.6%). Furthermore, a significant difference was observed between the dominant and non-dominant legs in unilateral 1RM performance. Together, these distinct differences confirm that lower-limb loading dynamics are task-specific and that traditional bilateral testing can mask true underlying limb imbalances.

The split-load BDR of 14.29% falls just below the commonly cited 15% threshold often used to designate “clinical” or “injury risk” asymmetry [26]. Conversely, the traditional bilateral leg press yielded a BSA of only 5.17%, which would be classified as “normal” or symmetrical under most screening guidelines. The presence of a greater BLD in the split-load leg press (14.29%) compared to the bilateral leg press (5.16%) aligns with prior findings that BLD becomes more pronounced in tasks requiring independent limb control [27]. Exercises such as the leg press, squat, or vertical jump, especially when performed unilaterally or in split stance, often expose hidden asymmetries that bilateral tasks can mask due to compensatory strategies [11]. BLD arises from a combination of neural and biomechanical factors, including reduced motor unit recruitment during bilateral tasks, poor interlimb coordination, and imbalances in limb-specific motor control pathways [27]. Additionally, prior musculoskeletal injuries, especially involving the knee, hip, or ankle, can result in persistent limb strength discrepancies—even years after rehabilitation [7]. Functional asymmetries have been strongly linked to increased risk of reinjury and performance deficits in sprinting, jumping, and cutting tasks [5,6].

However, the use of a fixed 15% threshold should be interpreted with caution. Current evidence suggests that inter-limb asymmetry is highly task-specific and exhibits substantial variability across tests and individuals, limiting the applicability of universal cut-off values [26,28]. From this perspective, the discrepancy observed between bilateral and split-load conditions may not necessarily indicate a “false negative” in the traditional bilateral task but rather reflect the context-dependent expression of asymmetry. Nevertheless, the split-load configuration appears more sensitive to detecting imbalances when limbs contribute independently, which may be advantageous in identifying underlying neuromuscular differences that are otherwise masked during bilateral execution. Given that asymmetries exceeding ~15% have been associated with increased injury risk in some prospective studies [1,26], the split-load leg press may offer greater sensitivity for identifying potentially meaningful deficits at a single time point being supported by significant difference between split-load and bilateral condition. However, due to the cross-sectional nature of the present study and the known variability in asymmetry thresholds, these findings should be considered preliminary and interpreted cautiously, particularly when translating them into practical or clinical decision-making.

The study revealed strong correlation between BAI for knee flexion showing significant negative correlations with the hamstring-to-quadriceps (H/Q) ratio of the dominant leg (*r* = −0.61, *p* < 0.01) and moderate correlation for non-dominant H/Q ratios (*r* = 0.37). While isokinetic dynamometry is considered the “gold standard” for strength assessment, it isolates muscle groups in an open kinetic chain, which may not fully transfer to closed kinetic chain functional movements like the leg press. The shared variance between these tests is consistent with previous literature, such as Impellizzeri et al., who found that open-chain knee extension tests explained only a portion of the variance in vertical jump performance [29].

Interestingly, BAI for knee flexion was moderately correlated with both BSA and BAI in the leg press (*r* = 0.40 and *r* = 0.39, respectively). This relationship was not observed for knee extension variables, suggesting that knee flexors may contribute to inter-limb asymmetries during the leg press. Although the leg press is primarily driven by the knee extensors, previous research suggests that the hamstrings play an important role in joint stabilization and neuromuscular coordination during multi-joint movements. Consequently, the observed associations may reflect the contribution of the knee flexors to inter-limb coordination and asymmetry, rather than their involvement in force production per se [30].

A critical methodological finding was the significant main effect of condition on BSA values (F(4,120) = 6.43, *p* = 0.0001), whereas the BAI did not differ significantly across conditions (F(4,120) = 1.23, *p* = 0.303). This highlights the “calculation conundrum” present in asymmetry research [28]. The BSA metric utilizes a “stronger vs. weaker” limb comparison, which yielded values ranging from 5.17 ± 4.44% in the leg press to as high as 17.84 ± 12.40% in eccentric hip extension. In contrast, BAI (using dominant vs. non-dominant distinctions) remained lower, with leg press values of only 2.27 ± 2.80%. As observed in this study and others, the dominant limb is not consistently the stronger limb across all tasks. Parkinson et al. argue that indices utilizing a stronger/weaker comparison avoid the limitations associated with arbitrary reference limbs, potentially explaining why BSA was more sensitive to condition changes in the current study than BAI [31].

Our study incorporated isokinetic measurements to validate the leg press as a reliable method for assessing BLD. While our study focused on a dynamic contraction model, Botton et al. demonstrated a similar magnitude of BLD between isometric and concentric knee extensions, suggesting that the phenomenon is not necessarily dependent on contraction type [32]. Despite ongoing investigation, the mechanisms underlying BLD remain complex and multifactorial. Four primary factors are believed to contribute to BLD. First, psychological factors may influence BLD, particularly due to differences in perceived exertion between unilateral and bilateral tasks. Second, BLD is highly task-specific and influenced by the degrees of freedom and stability requirements of the movement. We addressed this in our study by including a familiarization session, which helped minimize the influence of learning effects. However, BLD can still vary based on exercise selection. Second, biomechanical factors such as trunk stability and the use of counterbalances also influence BLD. Hirano et al. highlighted how both isokinetic knee flexion and extension are affected by trunk efficiency and joint stabilization, supporting the comparability of these methods in assessing BLD [33]. Lastly, physiological and neurophysiological factors play critical roles. These include the contribution of synergist and antagonist muscles, motor unit recruitment patterns, muscle fiber inhibition, spinal mechanisms, and higher-order neural inhibition, all of which may contribute to variations in electromyographic activity and overall force production [27].

BLD is influenced by the type of sport-specific training, thus, requiring a different approach for resolving it. If we focus on athletes that demand explosive effort, it is better to treat BLD with unilateral plyometric training rather than unilateral training [34]. Whereas for other strength training, bilateral patterns have the best outcomes to reduce BLD [35]. However, longitudinal studies often overlook the influence of cross-education, the phenomenon where unilateral strength training leads to strength gains in the untrained, opposite limb. This effect can potentially confound or mask the true impact of training interventions on bilateral deficit, particularly when unilateral exercises are used [36].

For instance, unilateral resistance training has been shown to produce superior adaptations in unilateral strength and neuromuscular control compared to bilateral training, making it particularly useful for addressing asymmetry-related deficits [37]. Incorporating eccentric-focused training—such as Nordic hamstring curls or eccentric step-downs—enhances neuromuscular control and reduces asymmetry, particularly in muscle groups responsible for deceleration and joint stability. Eccentric training has been shown to stimulate high levels of muscle activation and positively influence the hamstring-to-quadriceps (H/Q) ratio, which is essential for knee health and anterior cruciate ligament (ACL) injury prevention [38]. Additionally, neuromuscular training, including proprioception drills, dynamic balance work, and plyometrics, can reestablish symmetrical movement patterns and improve interlimb coordination. Using tools like biofeedback, force plates, or real-time motion tracking further enhances training specificity by allowing athletes to visually and kinesthetically correct asymmetrical outputs.

The relationship between BLD and athletic performance or injury risk remains unclear. Bračič et al. examined BLD in the countermovement jump and its association with sprint-start performance in elite sprinters [39]. Their findings indicated that lower BLD values were linked to greater peak force production by the rear leg during a double-leg sprint start, as well as a higher total force impulse on the starting blocks. In contrast, Bishop et al. reported that a greater BLD was associated with improved change in direction speed but not with linear sprinting speed [40]. These contrasting findings emphasize the importance of tailoring training strategies based on the specific performance demands of each sport. While the impact of BLD on injury risk remains underexplored, Andrade et al. found that patients exhibited high bilateral deficits six months after ACL reconstruction, which appeared to be associated with a higher rate of subsequent knee injuries and a reduction in Tegner activity levels [41].

### Limitations 

It should be acknowledged that asymmetry is not solely a magnitude issue; the direction of asymmetry (which limb is stronger) can fluctuate between tasks [26]. While this study utilized the BSA to quantify magnitude, future analyses should consider monitoring the direction of asymmetry over time, as consistent dominance in one limb across multiple tasks may pose a different risk profile than fluctuating dominance. Additionally, while isokinetic tests revealed significant asymmetries in knee muscles, hip extension did not, suggesting that the leg press BLD may be driven predominantly by knee extensor deficits in this specific cohort. Furthermore, the relatively small sample size may limit the generalizability of the findings and reduce the statistical power to detect smaller effects, particularly in variables where no significant differences were observed.

## 5. Conclusions

Split-load leg press might be used to test lower limb bilateral deficit, because it provides easily detectable deficit values. Unilateral leg press might be used to detect lateral strength differences, since it provides the relation to isokinetic strength of knee flexors and extensors. The observed BLD values are consistent with existing literature and highlight neuromuscular asymmetries that may not be detectable through traditional bilateral assessments. However, given the cross-sectional design, these findings should be interpreted with caution when considering their practical and clinical implications.

Future research should aim to validate these findings in larger and more diverse populations and, importantly, through longitudinal designs to determine whether asymmetries identified via split-load testing are predictive of injury risk or performance outcomes. Additionally, studies exploring the effectiveness of targeted interventions—such as unilateral training or neuromuscular re-education—on reducing these asymmetries and improving functional performance are warranted.

Overall, while split-load leg press testing appears to be a promising tool for asymmetry detection, its role in athlete monitoring, return-to-play decision-making, and long-term injury prevention strategies requires further empirical support.

## Figures and Tables

**Table 1:** Pairwise Comparisons of Bilateral Strength Asymmetry (BSA, %) Across Leg Press and Isokinetic Muscle Groups (Bonferroni-adjusted).

Comparison	Mean Difference	*p*-Value	95% Confidence Interval Lower Bound	95% Confidence Interval Upper Bound
Leg press BSA (%) vs. Knee flexors BSA (%)	−7.63	0.002	−13.14	−2.12
Leg press BSA (%) vs. Knee extensors BSA (%)	−4.56	0.34	−10.78	1.66
Leg press BSA (%) vs. Hip extensions concentric BSA (%)	−6.7	0.022	−12.77	−0.064
Leg press BSA (%) vs. Hip extensions eccentric BSA (%)	−12.67	<0.001	−20.33	−5.01
Knee flexors BSA (%) vs. Knee extensors BSA (%)	3.07	1	−5.62	11.76
Knee flexors BSA (%) vs. Hip extensions concentric BSA (%)	0.93	1	−7.28	9.129
Knee flexors BSA (%) vs. Hip extensions eccentric BSA (%)	−5.04	1	−14.68	4.6
Knee extensors BSA (%) vs. Hip extensions concentric BSA (%)	−2.15	1	−8.92	4.63
Knee extensors BSA (%) vs. Hip extensions eccentric BSA (%)	−8.11	0.068	−16.56	0.34
Hip extensions concentric BSA (%) vs. Hip extensions eccentric BSA (%)	−5.97	0.68	−15.5	3.57

**Table 2:** Inter-limb ratio results.

	Mean ± SD	Min	Max	95% CI
Relative bilateral leg press	3.96 ± 0.78	2.67	5.81	0.29
Relative split-load leg press	3.54 ± 0.75	2.31	5.58	0.27
Relative unilateral leg press dominant	2.13 ± 0.42	1.45	3.26	0.15
Relative unilateral leg press non-dominant	2.03 ± 0.36	1.39	2.85	0.13
H/Q dominant	0.54 ± 0.1	−0.39	0.75	0.27
H/Q non-dominant	0.54 ± 0.11	−0.29	0.71	0.25
Leg press BSA (%)	5.17 ± 4.44	0	14.29	1.63
Knee extensors BSA (%)	9.73 ± 9.85	0	38.46	3.61
Knee flexors BSA (%)	12.8 ± 10.46	0	41.75	3.84
Hip extensions eccentric BSA (%)	17.84 ± 12.4	1	42.58	4.55
Hip extensions concentric BSA (%)	11.87 ± 9.99	0.49	36.39	3.67
Leg press BAI (%)	2.27 ± 2.8	−2.91	7.69	1.03
Knee extensors BAI (%)	3.2 ± 7.38	−12.71	23.81	2.71
Knee flexors BAI (%)	3.32 ± 9.03	−17.27	26.38	3.31
Hip extensions eccentric BAI (%)	4.32 ± 12.28	−20.62	27.05	4.5
Hip extensions concentric BAI (%)	0.05 ± 8.99	−22.24	16.67	3.3
Bilateral deficit ratio—Leg press split-load (%)	14.29 ± 7.71	−1.69	32.43	11.42
Bilateral deficit ratio—Leg press bilateral (%)	5.16 ± 9.6	23.81	24.14	8.50

**Table 3:** Correlation table.

	H/Q Dominant	H/Q Non-Dominant	Non-Dominant Flexors/Dominant Extensors Ratio	Dominant Flexors/Non-Dominant Extensors Ratio	BDR Leg Press Split Load	BDR Leg Press Bilateral	BSA Leg Press	BAI Leg Press
BSA Knee Extension (%)	−0.04	−0.53 **	−0.18	−0.12	0.01	−0.18	−0.16	−0.3
BSA Knee Flexion (%)	−0.38 *	0.15	0	0.03	0.15	0	0.29	0.33
BSA Hip Extension Concentric (%)	0.08	−0.06	−0.26	−0.15	−0.28	−0.26	−0.05	−0.2
BSA Hip Extension Eccentric (%)	0.05	−0.25	0.23	−0.13	0.05	0.23	−0.22	−0.07
BSA Leg press (%)	−0.22	0.32	−0.05	0.19	−0.03	−0.05	-	0.87 **
BAI Knee Extension (%)	0.06	−0.63 **	−0.03	0.03	0.13	−0.03	−0.22	−0.12
BAI Knee Flexion (%)	−0.61 **	0.37 *	−0.05	0.42 *	0.07	−0.05	0.40 *	0.39 *
BAI Hip Extension Concentric (%)	−0.36 *	−0.26	0.02	−0.27	0.23	0.02	0.21	0.34
BAI Hip Extension Eccentric (%)	−0.01	−0.1	0.06	0.09	0.14	0.06	−0.03	−0.02
BAI Leg Press (%)	−0.23	0.23	0.07	0.18	0.09	0.07	0.87 **	-

* Correlation is significant at the 0.05 level (2-tailed). ** Correlation is significant at the 0.01 level (2-tailed). BSA—bilateral strength asymmetry; BAI—bilateral asymmetry index, H/Q—hamstring to quadriceps ratio, BDR—bilateral deficit ratio.

## Data Availability

The data are not publicly available due to privacy.

## Supplementary Materials

The following supporting information can be downloaded at: https://www.mdpi.com/article/10.3390/jfmk11020216/s1.

## Abbreviations

The following abbreviations are used in this manuscript:

BDR	bilateral deficit ratio
BSA	bilateral strength asymmetry
BAI	bilateral asymmetry index
1RM	one-repetition maximum
H/Q	hamstrings-to-quadriceps
ACL	anterior cruciate ligament
