# Supplementary figures and images for: Hip and Knee Bilateral Deficit Across Bilateral, Unilateral, and Split-Load Leg Press Conditions
Source: J Funct Morphol Kinesiol. 2026 May 28;11(2):216. doi: 10.3390/jfmk11020216 (PMC13301863; doi:10.3390/jfmk11020216)

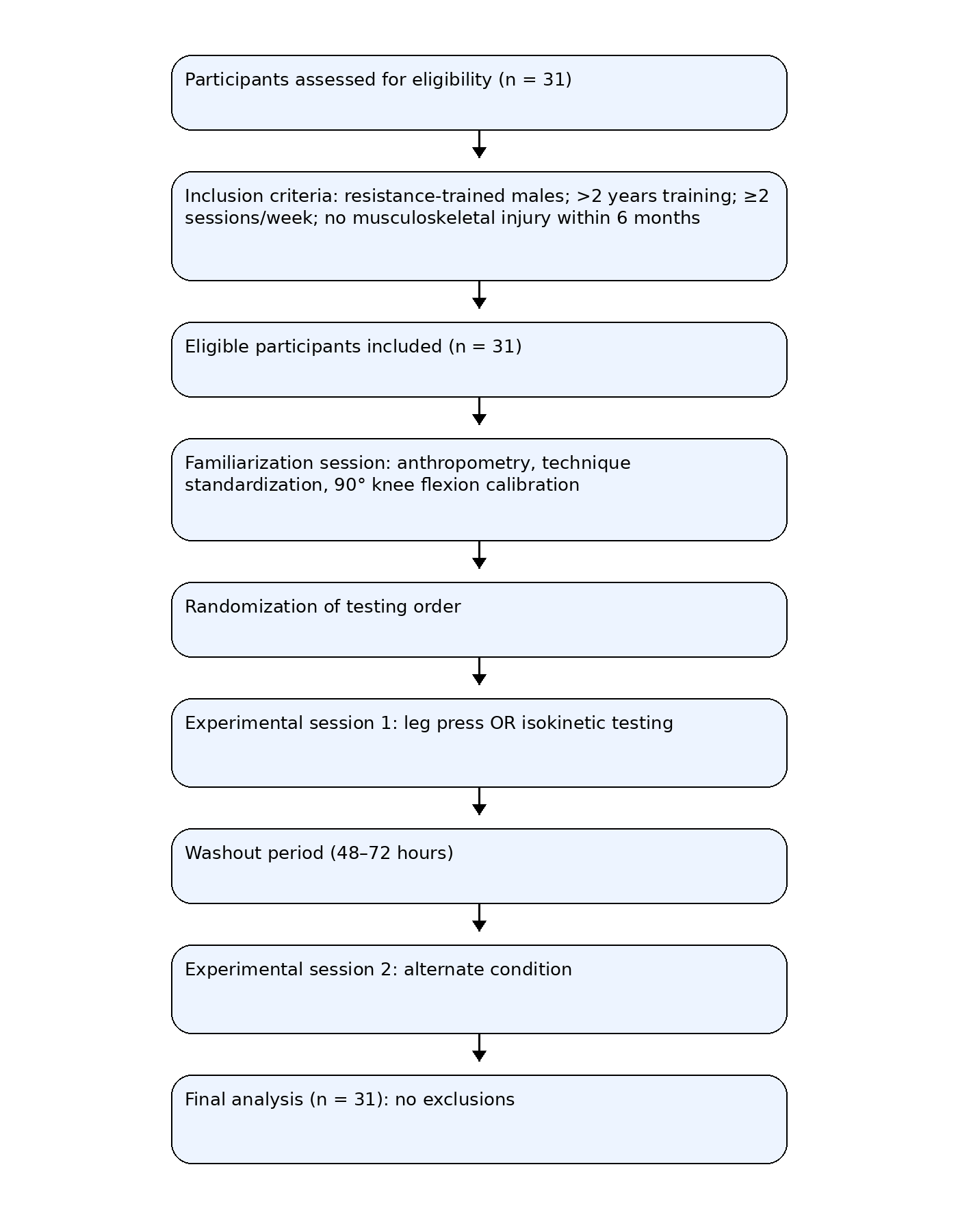

Supplement: Supplementary file 1 [file jfmk-11-00216-s001.zip › jfmk-4272901-supplementary.png]
